# Horner’s Syndrome Following Thyroid Surgery

**DOI:** 10.7759/cureus.45825

**Published:** 2023-09-23

**Authors:** Abdulaziz A Arishi, Farouk Abualhana, Joseph Sferra

**Affiliations:** 1 General Surgery, Faculty of Medicine, Jazan University, Jazan, SAU; 2 General Surgery, The University of Toledo College of Medicine and Life Sciences, Toledo, USA; 3 Internal Medicine, The Ohio State University College of Medicine, Columbus, USA; 4 General Surgery, ProMedica Toledo Hospital, Toledo, USA

**Keywords:** eye ptosis, miosis, post-thyroidectomy complication, papillary thyroid cancer, thyroid anatomy, horner’s syndrome

## Abstract

Horner’s syndrome (HS) is a rare complication of thyroidectomy caused by damage to the oculosympathetic nerves. This article reports the case of a 29-year-old woman referred to the clinic with a newly diagnosed papillary thyroid carcinoma (PTC). Ultrasound studies were concerning for multiple thyroid nodules and an enlarged lymph node, confirmed by a computed tomography (CT) scan. Cytology results of fine needle aspiration (FNA) diagnostic for PTC showed tumors in the thyroid tissue and lymph node. The patient underwent a thyroidectomy and woke up with right-sided ptosis and miosis. Clinical follow-up revealed subjective ipsilateral anhidrosis. She also developed a low parathyroid hormone level and dysphonia, albeit they resolved after months. The patient still exhibits HS eight months after surgery. This paper reviews the literature and attempts to establish the most probable causal factor while providing implications for surgical teams to minimize HS occurrence in future thyroid surgeries.

## Introduction

Horner's syndrome (HS) is a disorder that paralyzes the eyes and surrounding facial muscles due to partial or complete disruption of the central nervous pathways originating from the brain to the face. It was first described by Swiss ophthalmologist J.F. Horner and has since become a topic of interest in surgical care. HS occurs when the oculosympathetic pathway is disrupted [1]. The disease is characterized by anomalies or malfunctioning of facial organs. Clinically, it manifests through "miosis, ipsilateral blepharoptosis, enophthalmos, facial anhidrosis, and vascular dilation of the lateral part of the face" [1].

HS is a rare complication of thyroid diseases. The majority of etiologies involve compression of the cervical plexus. Thyroid carcinoma accounts for 21% of cases. Among reported cases, multinodular goiter, Riedel's and Hashimoto's thyroiditis, thyroid adenoma, thyroid lymphoma, sympathetic paraganglioma, neuroblastoma, schwannoma, and Ewing sarcoma have been associated with HS. Most of these cases have occurred in an adult population.

HS can also arise for various reasons, including trauma, apical lung tumors, or carotid artery dissection, but thyroid surgery (thyroidectomy) is an increasingly concerning causal factor, which could surgically damage the nerve pathway [2]. Horner syndrome following thyroid surgery is an extremely rare complication, with an incidence of less than 0.2% to 0.3% among patients undergoing thyroidectomy [3].

The disorder can be diagnosed through clinical observations by an ophthalmologist, who may also use drugs such as cocaine to confirm the disease. Cocaine blocks the reuptake of the neurotransmitter norepinephrine from the synaptic cleft. When instilled into the eyes, there will be pupillary dilation in the normal eye but not in the eye affected by HS.

This research paper reports a case of HS in a thyroidectomy patient referred to our clinic who underwent central neck dissection for papillary thyroid carcinoma (PTC). HS occurs as a rare complication of thyroid surgery due to the disruption of the oculosympathetic nerves and must be addressed, as its impact on patients is non-reversible.

## Case presentation

**Figure 1:**
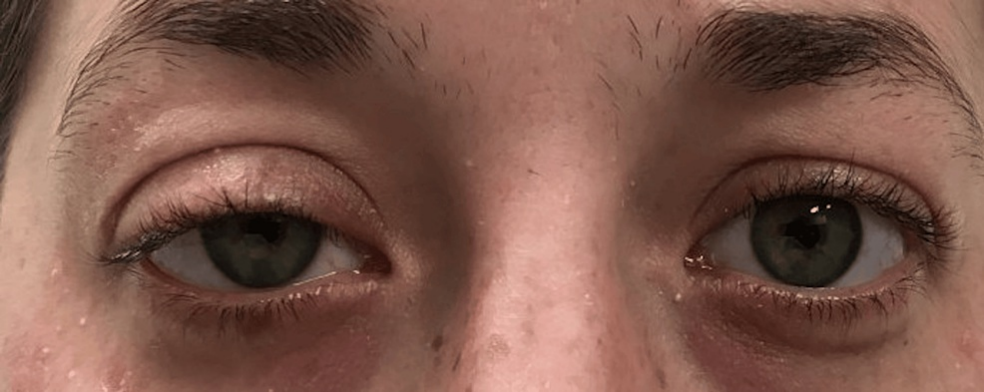
Patient with right-sided eye ptosis and miosis.

A 29-year-old woman was referred to our clinic with a newly diagnosed PTC. Ultrasound studies were concerning for multiple thyroid nodules and an enlarged lymph node. The cytology results of fine needle aspiration (FNA) confirmed PTC (Bethesda VI) in the thyroid tissue and lymph node. CT scan of the neck suggested the presence of enlarged lymph nodes in the central neck region. Intraoperatively, the superior and recurrent laryngeal nerves were identified and carefully dissected in conjunction with nerve stimulation monitoring. Stemming of the recurrent laryngeal nerve near the placement of a metal clip was noted, but the nerve did appear to recover function once the clip was removed. A tumor was noted adjacent to the carotid sheath and middle cervical ganglion, so careful retraction of the artery was performed for adequate exposure. Surgical pathology revealed resection of 23 lymph nodes, of which nine were positive for PTC. The report also identified the removal of a left inferior parathyroid gland and a right intrathyroidal parathyroid gland. The patient awoke with right-sided ptosis and miosis and, at clinic follow-up, reported subjective ipsilateral anhidrosis, and her immediate postoperative outcome was complicated by a low parathyroid hormone level and dysphonia (Figure 1).

These latter two complications both resolved over the following months. She continues to exhibit HS eight months after surgery.

## Discussion

Pathophysiology of HS

To understand the clinical manifestations of HS, it is imperative to briefly describe the anatomy of the oculosympathetic pathway (OSP). According to Kong et al., owing to its striking features, the diagnosis of HS is primarily clinical [4]. The OSP begins with first-order neuronal fibers from the hypothalamus that descend and terminate at the ciliospinal center (C8-T2). Second-order preganglionic fibers exit T1 and ascend the sympathetic chain and stellate ganglion to end in the superior cervical ganglion (SCG). Third-order postganglionic fibers exit the SCG to rise as a plexus along the walls of the ICA and then follow the ophthalmic branch of the trigeminal nerve to the orbit, supplying the dilator pupillae and superior tarsal muscle. Vasomotor and sudomotor gland fibers course separately, following the external carotid artery to the face. Interestingly, thyroid branches originate from the middle cervical ganglion and course with the inferior thyroid artery to anastomose with the superior cardiac, superior laryngeal, and recurrent laryngeal nerves [3]​​​​. Rare anatomic variations exist in which the recurrent laryngeal nerve gives off a communicating branch to the sympathetic chain. In such instances, nerve manipulation is a potential source of HS [5]. Overall, the anatomical proximity with the thyroid makes the sympathetic nerve pathway and the middle cervical ganglion susceptible to damage during surgery, mainly when conducted with central neck dissection for papillary thyroid carcinoma (PTC), as was the case in the patient in question.

HS may result from the direct interruption of the stellate ganglion or the first white ramus communicans or indirect interruption of the anastomoses mentioned above. HS post-thyroidectomy may involve any of the following: postoperative hematoma, lateral ligature of the inferior thyroid artery trunk inducing neural ischemia, stretching of the sympathetic chain by the retractor, and disturbance of the communication between the sympathetic chain and recurrent laryngeal nerve during its identification [3]. The prognosis of HS has been proposed to be poor depending on the particular mechanism of injury. However, if the injury is related to hematoma formation, inflammation, or peripheral ligature of the inferior thyroid artery branches, the HS may spontaneously resolve. In minimally invasive video-assisted thyroidectomy (MIVA), heat injury induced by the ultrasonic scalpel can damage the oculosympathetic pathway owing to its close anatomical proximity to the thyroid gland, leading to HS [6]. The same case applies to thyroid microwave ablation (MWA). According to Zhang et al., the high heat generated by the active tip of the electrode damages the sympathetic nerves by causing neural lesions that disrupt the normal communication and transfer of signals from the brain [7]. All these instances are rare. However, surgeons and healthcare providers must remain aware of these possibilities when considering a complex thyroid surgery.

The patient’s HS is likely to emanate from two factors. The acute onset of ptosis and miosis in the case patient suggests direct interruption of the sympathetic chain via stretching the carotid plexus by the retractor rather than a postoperative hematoma. Ideally, in an attempt to expose the thyroid tissue, the surgeon could have forcefully stretched the sympathetic trunk and destroyed it, leading to the loss of nerve communication. This surgical damage seems the most probable hypothesis for the patient’s HS. Another less likely yet plausible reason is that the patient had dysphonia after surgery, and therefore, there was the presence of an anatomically variant recurrent laryngeal nerve whose communicating branch to the sympathetic chain was already disrupted. Thus, the chances are high that the surgery only aggravated the situation by damaging the nerves, causing an acute onset of HS.

Given the close anatomical relationship between the thyroid and the oculosympathetic pathway, thyroid surgeries must be carefully approached. Giannaccare et al. recommend that patients scheduled for thyroid surgery undergo imaging first to accurately characterize the entire neck thyroid region and the positioning of the sympathetic trunk [2]. These suppositions are supported by Yeh et al., who affirm that imaging the neck as a preoperative procedure allows surgeons to locate abnormal lymph nodes that could obstruct the removal of the tumor [8]. Consequently, surgeons can identify a surgical site that offers the best access to the thyroid tumors while minimizing any significant infraction on the oculosympathetic nerve pathway. For instance, in the case under review, a CT scan on the patient revealed enlarged lymph nodes, which implied that the surgery would have been more complex. Enlarged lymph nodes denote that access to the thyroid and ganglion region was limited. Hence, the surgeon had to retract the tissues above to expose the thyroid gland laterally. While doing so, the mechanical retraction could have stretched the sympathetic chain and disrupted its anatomical integrity, resulting in the acute onset of HS immediately after the thyroid surgery.

Understanding clinical features of HS after surgery

Patient follow-up is critical to identifying HS onset and paves the way for immediate interventions following a thyroid surgery to remove a tumor. An evident clinical sign is miosis, which occurs immediately after the loss of sympathetic function and leads to the constriction of the eye’s pupil [4]. Therefore, part of postoperative care should include shining a bright and dim light on the patient’s eye to confirm normal functioning of the sympathetic nerve by observing pupil dilation. Additionally, following the disruption of the sympathetic nerves, patients could exhibit HS through partial ptosis and enophthalmos. This condition lowers the upper eyelid and elevates the lower lid to narrow the palpebral fissure [4]. Besides, the patient demonstrates ipsilateral anhidrosis, in which the face and neck become flushed and dry and can be evidenced on the ear lobes and in the conjunctiva [4]. Postoperative nurses and caregivers at home must be mindful of these clinical features of HS and report them immediately to facilitate early interventions to manage the condition.

## Conclusions

HS as a complication of thyroidectomy is an uncommon phenomenon. Knowing the causes and anatomic variations involved can help further reduce its occurrence. Surgical teams should be aware of the possibility of this rare but important complication. Patients with HS have to live with facial disfiguration and damage to the eyes due to the malfunctioning of the sympathetic nerves. The resolution is often incomplete for the few who recover, and a patient has to live with lasting anomalies. 
